# Supplementary material for: Bacterial co-culture with cell signaling translator and growth controller modules for autonomously regulated culture composition
Source: Nat Commun. 2019 Sep 11;10:4129. doi: 10.1038/s41467-019-12027-6 (PMC6739400; doi:10.1038/s41467-019-12027-6)
Supplement: Supplementary file 1 — Supplementary Information [file 41467_2019_12027_MOESM1_ESM.pdf]

Supplementary Information

"Bacterial co-culture with cell signaling translator and growth controller modules for autonomously regulated culture composition"

Stephens et al.

Supplementary Figures

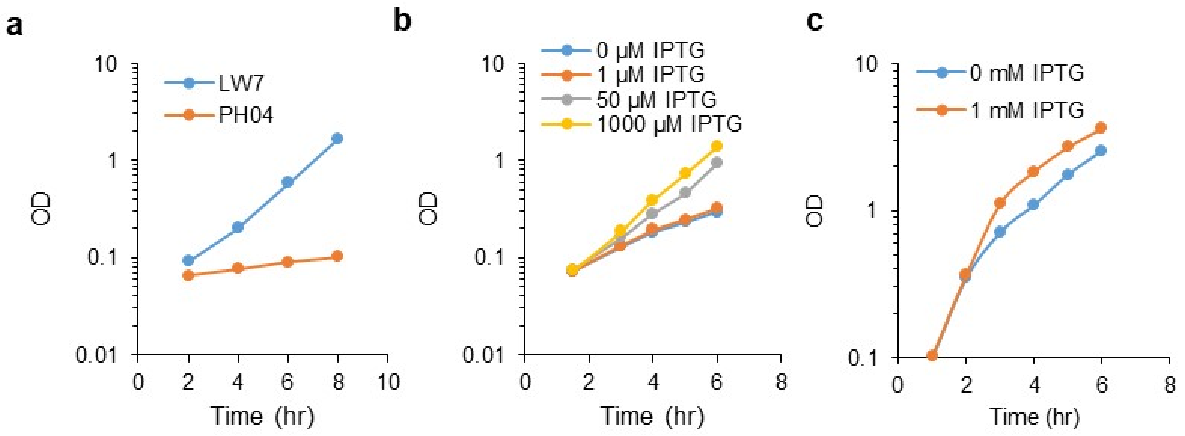

Supplementary Figure 1. Regulating cell growth rate through HPr. (**a**) Growth curves of LW7 (W3110 Δ*luxS* Δ*lacZ*) and PH04 (W3110 Δ*luxS* Δ*lacZ* Δ*ptsH*) in M9 media (0.8% glucose). (**b**) Growth curves of PH03 (W3110 Δ*lacZ* Δ*ptsH*) pTac-HPr (containing *ptsH* under *tac* inducible promoter) induced with varying levels of IPTG supplemented at $t = 0$. Cultures grown in M9 media (0.8% glucose, 0.2% casamino acids). (**c**) Growth curves of PH03 pTac-HPr in LB media with and without IPTG (induced at $t = 0$). One biological replicate is shown in each panel. Source data are provided as a Source Data file.

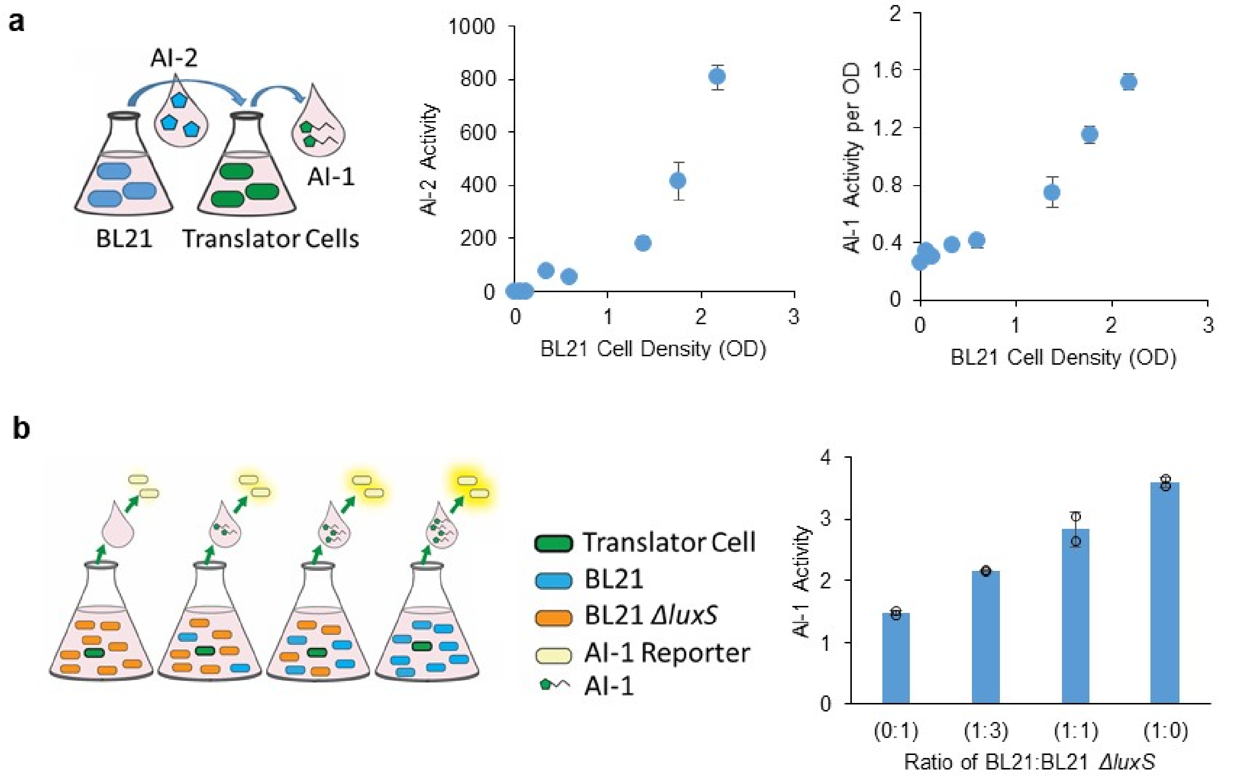

Supplementary Figure 2. AI-2 producing cells activate AI-1 synthesis in translator cells. (a) BL21 cells were grown in LB media. At various cell densities, CM samples were collected to measure AI-2 activity (left panel). CT104 pCT6 pLasI translator cultures were grown to OD 0.1, resuspended in CM from the BL21 cultures and AI-1 activity per translator cell OD was measured after five hours (right panel). Error bars represent s.d. of technical duplicates. **(b)** CT104 pCT6 pLasI translator cells were added to consortia composed of varying ratios of BL21 (produces AI-2) to BL21 *ΔluxS* (does not produce AI-2) in LB media. Combined BL21 and BL21 *ΔluxS* initial OD was approximately 0.25 and initial translator OD was 0.02. After 5 hours CM samples were taken to measure AI-1 activity. Error bars represent s.d. of technical duplicates. Source data are provided as a Source Data file.

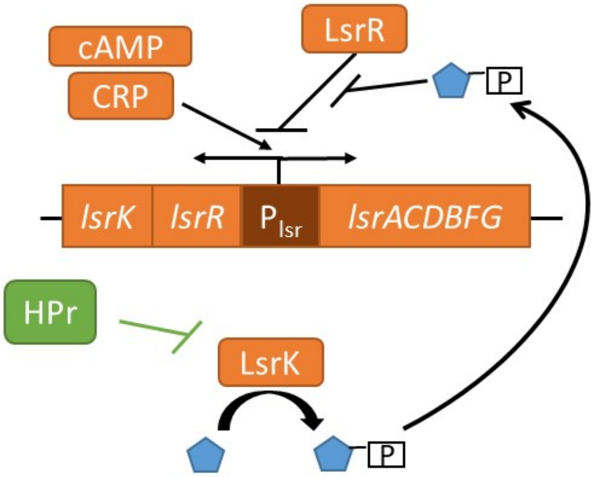

Supplementary Figure 3. Scheme of AI-2 pathway. In the native AI-2 quorum sensing system, LsrACDB transports AI-2 into the cell. LsrK phosphorylates AI-2, which subsequently causes derepression of the *lsr* promoter by LsrR and transcription of the *lsr* operon. This creates a positive feedback loop and additional uptake of AI-2. LsrFG degrade the phosphorylated AI-2 signal. The *lsr* promoter is also partially regulated by global regulators cAMP/CRP that are affected by the availability of glucose. In addition, the PTS protein HPr (involved in sugar transport into the cell) has been shown to interact with LsrK and inhibit LsrK activity[1].

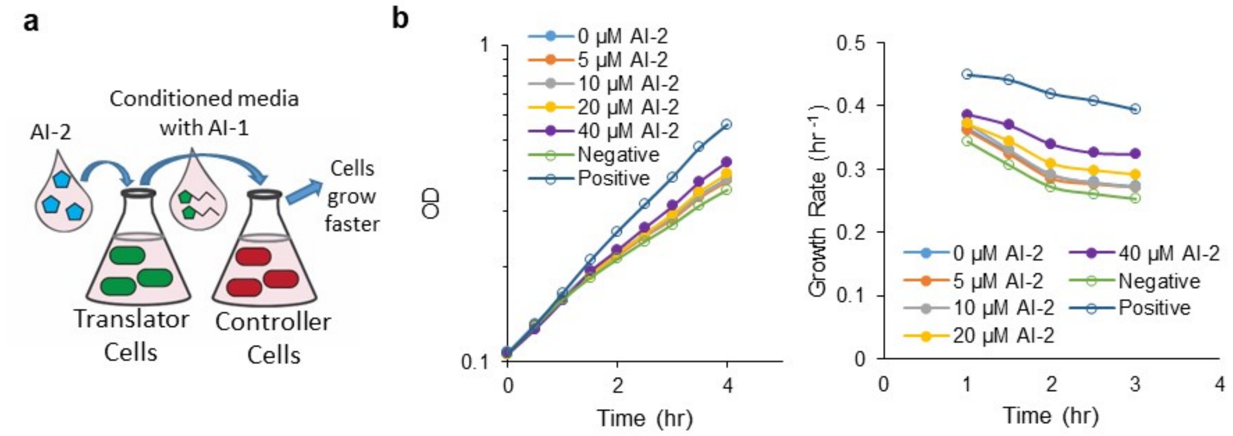

Supplementary Figure 4. Translator cells regulate controller cells based on initial AI-2 level. (**a**) Schematic of process for conditioned media experiments. Translator cells (PH04 pCT6 pLasI) are grown with varying levels of AI-2. Conditioned media from translator cells is added to controller cells (PH03 pAHL-HPr). (**b**) Growth curves and growth rates of controller cultures grown in M9 media supplemented with conditioned media from translator cells. Translator cells were grown for 3 hours with varying levels of AI-2 as indicated. Conditioned media samples from translator cultures were added to controller cultures at a 5% final volume at $t = 0$. The negative and positive controls used conditioned media from cells not capable of producing AI-1. For the positive control, 2 µM AI-1 was added at $t = 0$. One biological replicate is shown. Source data are provided as a Source Data file.

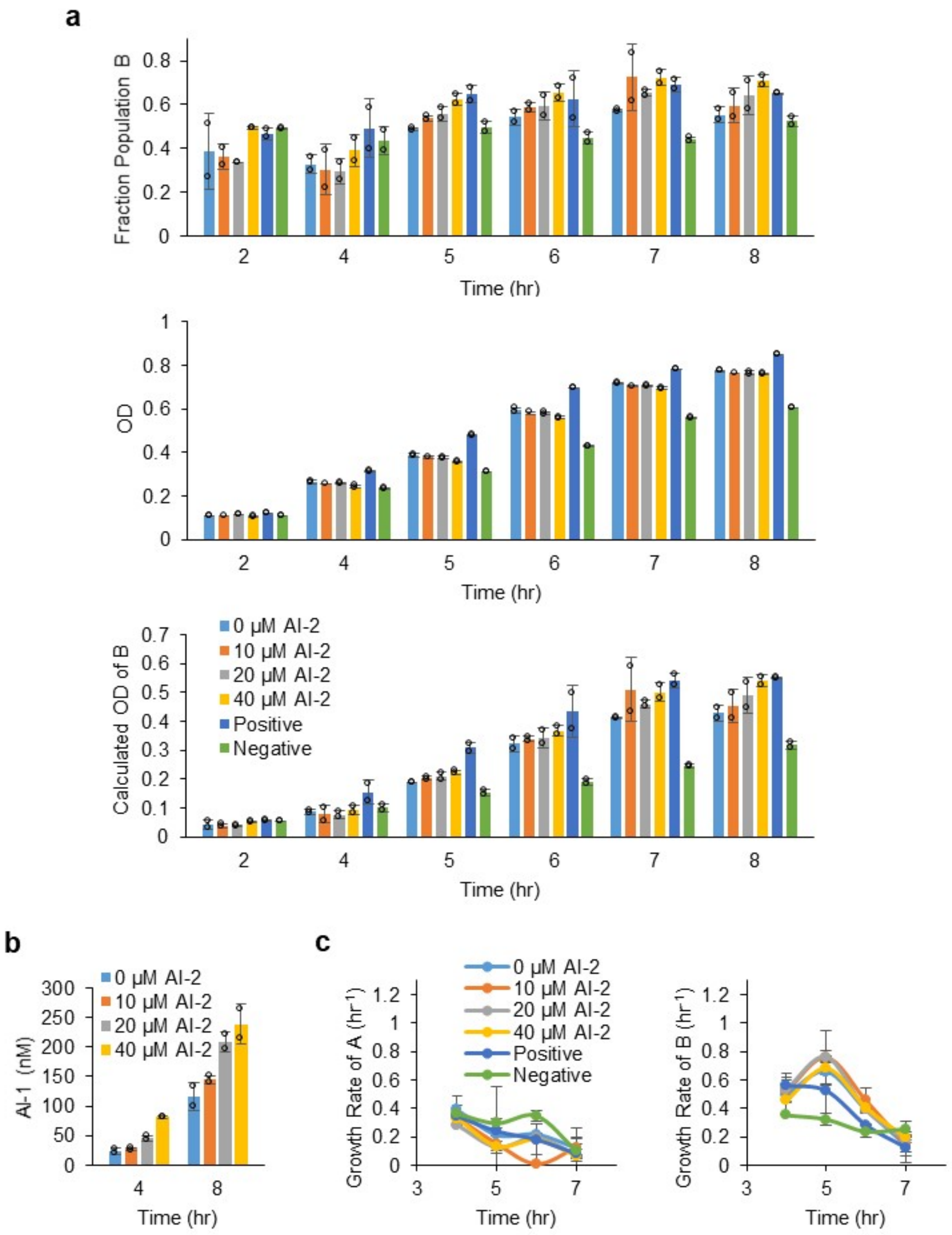

Supplementary Figure 5. Translator cells alter co-culture composition based on AI-2 level. (**a**) Translator cells (Population A, PH04 pCT6 pLasI) and controller cells (Population B, PH04 pAHL-HPr) were co-cultured in M9 media with varying levels of AI-2. Population B fraction, total culture density, and calculated OD of Population B over time are shown. Controller cells and translator cells were each inoculated 0.5% from overnight cultures and in media with AI-2 at $t = 0$. For the positive control 1 µM AI-1 was added at the start of the culture. The negative control used PH04 pAHL-sfGFP in place of controller cells. (**b**) Extracellular AI-1 level of co-culture. (**c**) Translator cell and controller cell growth rate in the co-culture. To determine the translator cell density in order to calculate growth rate, the red cell OD was subtracted from the total OD. Error bars represent s.d. between biological duplicates. Source data are provided as a Source Data file.

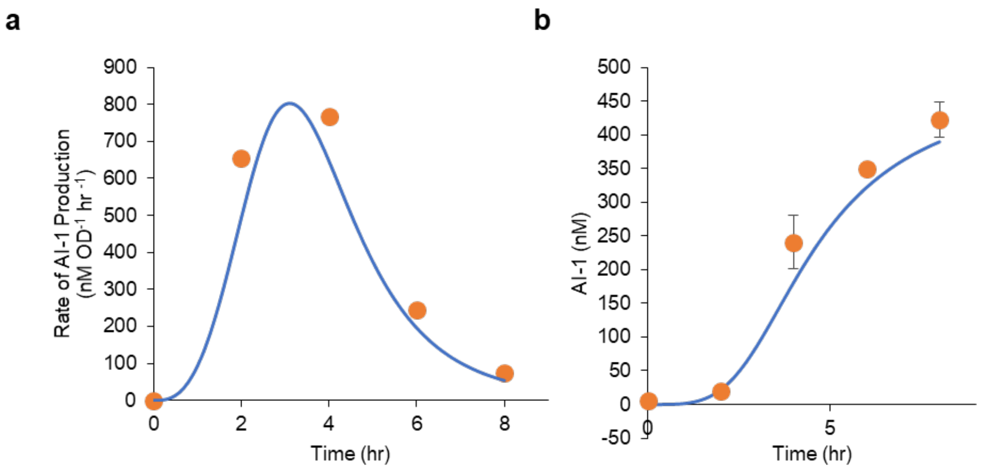

Supplementary Figure 6. Predicting translator cell behavior from co-culture data. The rate of AI-1 production by the translator cells for an 80 µM AI-2 addition was predicted using co-culture data from Figure 6. To confirm the prediction, translator cells were inoculated to approximately OD 0.05 from overnight cultures in M9 media with 80 µM AI-2 at $t = 0$. **(a)** The blue line shows the predicted *fAI1* function. The predicted function has the form shown for the *fAI1* functions in Supplementary Table S2, where $A = 0$, $B = 4$, $C = 3.6$, $D = 570$, and $E = 0.5$. The dots show the rate of AI-1 production during the translator monoculture experiment. (**b**) The orange dots show the AI-1 level in the translator monoculture with 80 µM AI-2 over time. The blue line shows the simulation results for a translator cell culture using the predicted *fAI1* function. Error bars represent s.d. of technical duplicates. Source data are provided as a Source Data file.

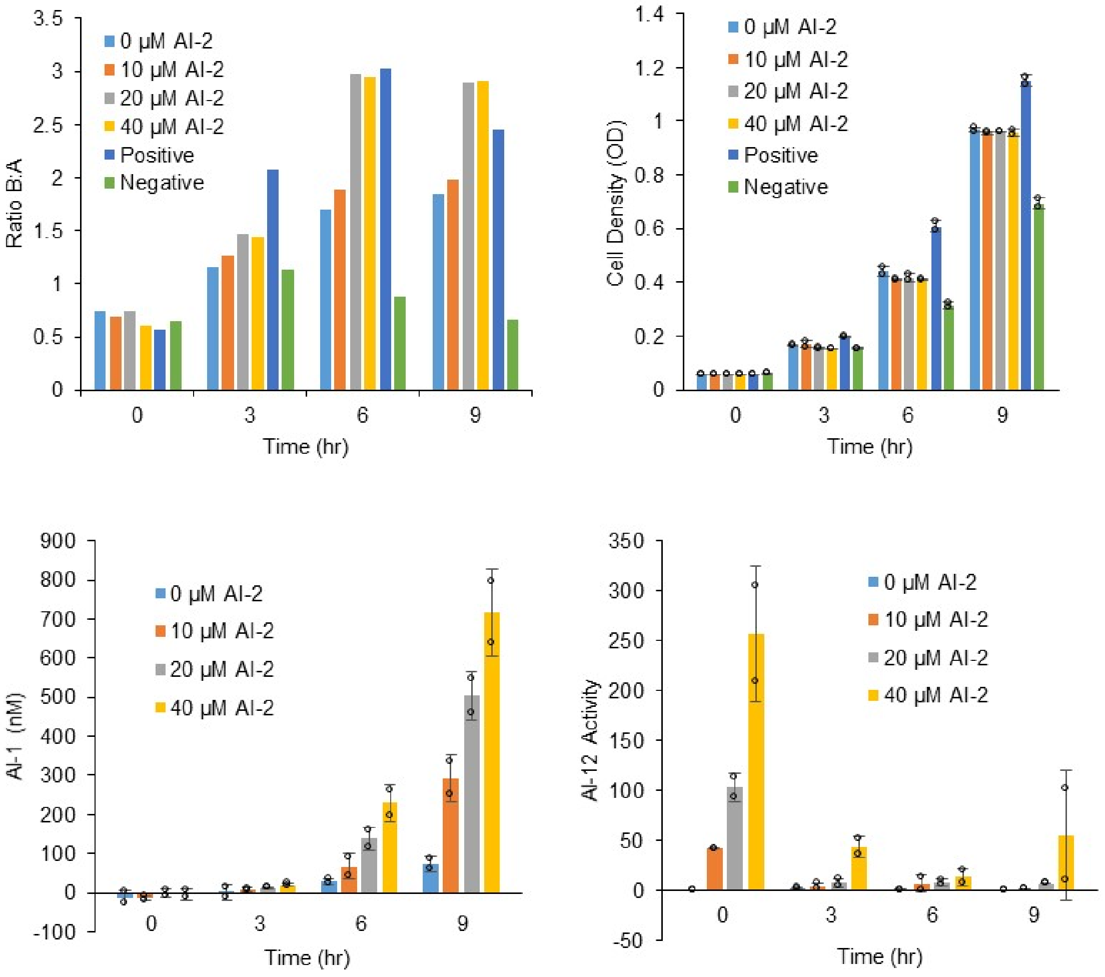

Supplementary Figure 7. Extended co-cultures of translator and controller cells. Populations A and B were each inoculated 0.5% with varying concentrations of AI-2 as indicated. For the positive control, 200 nM AI-1 was added in place AI-2. For the negative control, the AI-1 responsive cells were replaced with a cell line containing sfGFP in place of *ptsH* (PH04 pAHL-sfGFP). Every three hours cultures were spun down and resuspended in fresh media with either AI-2 or AI-1. Samples were collected at $t = 0$ and immediately prior to each resuspension for measurement of fraction Population B and cell density. Ratio of B:A was calculated using the average fraction Population B measurement. CM from experimental cultures were also collected for AI-1 and AI-2 activity measurements. Error bars represent s.d. of biological duplicates. Source data are provided as a Source Data file.

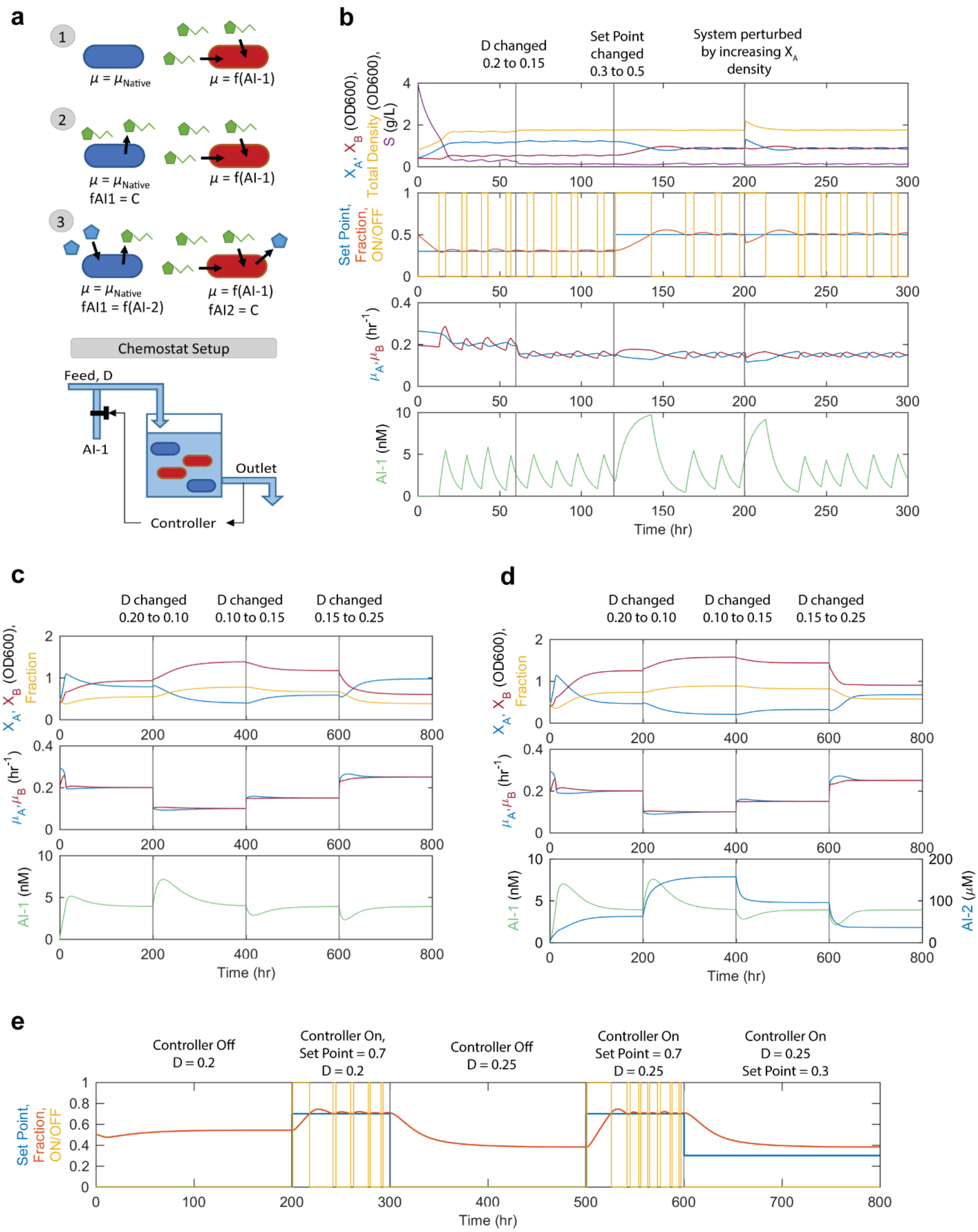
a
b
c
d
e
μ = μNative
μ = f(AI-1)
fAI1 = C
fAI1 = f(AI-2)
fAI2 = C
Chemostat Setup
Feed, D
AI-1
Outlet
Controller
D changed 0.2 to 0.15
Set Point changed 0.3 to 0.5
System perturbed by increasing XA density
XA, XB (OD600), Total Density (OD600), S (g/L)
Set Point, Fraction, ON/OFF
μA, μB (hr-1)
AI-1 (nM)
AI-2 (μM)
Time (hr)
D changed 0.20 to 0.10
D changed 0.10 to 0.15
D changed 0.15 to 0.25
XA, XB (OD600), Fraction
Controller Off D = 0.2
Controller On, Set Point = 0.7 D = 0.2
Controller Off D = 0.25
Controller On Set Point = 0.7 D = 0.25
Controller On D = 0.25 Set Point = 0.3

Supplementary Figure 8. Chemostat simulations using various populations and control schemes. a) Scheme depicting various co-culture designs consisting of Populations A (blue) and B (red). Each population produces QS signals as indicated. The growth rate of Population A is not controlled, but the growth rate of Population B is modulated by AI-1. The co-culture behavior is modeled in a chemostat with a continuous feed into and removal of culture from the reactor set by the dilution rate, D. Each co-culture set can be simulated with a control scheme where the culture composition is sampled hourly, compared to a set point, and a fixed concentration of AI-1 is either added or not to the feed. **b)** Model simulation of co-culture set 1 in a chemostat with the controller. An initial Set Point of 0.7 (fraction of Population B) is simulated. The controller adds or stops adding AI-1 to the feed (ON/OFF, where ON = 1 and OFF = 0) to maintain the desired set point. At the times indicated, the dilution rate is changed, the set point is changed, or the system is perturbed by simulating a sudden increase in Population A density. **c)** Simulation of co-culture set 2 in a chemostat without the "controller." Over time the system reaches steady state and a specific culture composition. Changing the dilution rate changes the steady state culture composition. **d**) Simulation of co-culture set 3 in a chemostat without the controller. **e)** Simulation of co-culture set 2 with the controller. As in (**c**), a steady state culture composition is reached based on the dilution rate. Then, a set point can be programmed to control the composition at a higher (but not a lower) level than the system would naturally achieve. See Supplementary Note 3 for further details on the model.

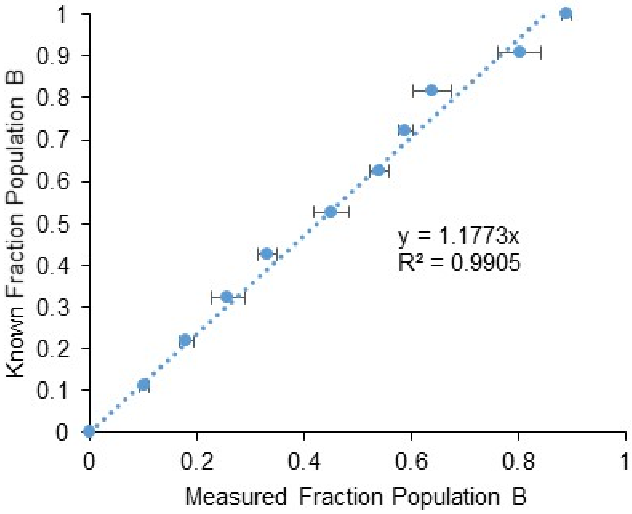

Supplementary Figure 9. Standard curve for measurement of fraction Population B. Translator cells and controller cells (expressing constitutive dsRedExpress2) were each inoculated 1% from overnight cultures and grown as monocultures in M9 media for five hours. After five hours, OD was recorded, and used to create co-cultures of known compositions. Microscopy and ImageJ were used to determine measured compositions. Error bars show s.d. between technical quadruplicates. Source data are provided as a Source Data file.

Supplementary Tables

Supplementary Table 1: Function for AI-1 production in translator cells.

**Equation**	**Description**
$Extracellular\ AI1 = D + \frac{A - D}{\left(1 + \left(\frac{t}{C}\right)^{B}\right)^{E}}$	Fits experimental results (Figure 5a) for translator culture extracellular AI-1 levels over time for different initial AI-2 concentrations.
$Cell\ Density = EXP(0.28t - 2.89)$	Fits experimental results (Figure 5c) for translator culture cell density over time.
$fAI1(t, AI2) = \frac{\frac{d}{dt}(Extracellular\ AI1(t))}{EXP(0.28t - 2.89)}$	AI-1 produced by translator cells over time in response to initial AI-2 level.

Where

fAI1	Rate of AI-1 produced (nM AI-1/(hr*OD))
t	Time (hr)
A, B, C, D, E	Constants dependent on initial AI-2 concentration (see below)

	**0 µM**	**10 µM**	**20 µM**	**40 µM**
A	0	0	0	0
B	4.4	3.5	3.5	3.8
C	6.4	9	6.48	4.40
D	295	300	350	505
E	0.83	2.75	1.19	0.49

Supplementary Table 2: System of ordinary differential equations used to model co-culture system.

Reaction	Differential Equation
Strain A Density	$\frac{dX_A}{dt} = \frac{\mu_{b\_A} * S}{K_A + S} * X_A$
Strain B Density	$\frac{dX_B}{dt} = \frac{S}{K_B + S} * \mu_{b\_B} * fHPr(AI1) * X_B$
Substrate Concentration	$\frac{dS}{dt} = -\frac{1}{Y_A}\frac{\mu_{b_A} * S}{K_A + S} * X_A - \frac{1}{Y_B}\frac{S}{K_B + S} * \mu_{b\_B} * fHPr(AI1) * X_B$
AI-1 Concentration	$\frac{dAI1}{dt} = fAI1(AI2) * X_A$
**Where**	
Function for increased growth rate (caused by HPr) in Population B	$fHPr(AI1) = D + \frac{A - D}{\left(1 + \left(\frac{AI1}{C}\right)^B\right)^E}$ Where $A = 1$, $B = 1$, $C = 29$, $D = 2.1$, $E = 0.48$
Function for production of AI-1 in Population A as a function of AI-2 and time from AI-2 addition	$fAI1(t, AI2)$ Varies depending on initial AI-2 concentration. See Table S1 and Figure 2d.

Supplementary Table 3: Initial conditions and constants used in model.

Species	Description	
X_A	Population A concentration (OD600)	
X_B	Population B concentration (OD600)	
S	Substrate Concentration (g/L)	
$AI1$	AI-1 Concentration (nM)	
$AI2$	Supplemented AI-2 Concentration (μM)	
S_0	Initial substrate conc. (8 g/L for media used)	
t	Time (hr)	
**Parameter**	**Description**	**Value**
μ_{b_A}	Basal specific growth rate of Population A (hr^{-1})	0.28
μ_{b_B}	Basal specific growth rate of Population B (hr^{-1})	0.32
K_A & K_B	Substrate concentration for half max growth (g/L)	0.1
Y_A & Y_B	Yield (OD600 cells)/(g/L substrate)	0.45

Supplementary Table 4: System of ordinary differential equations used in chemostat simulations.

Reaction	Differential Equation	
Strain A Density	$\frac{dX_A}{dt} = -D * X_A + \frac{\mu_{b\_A} * S}{K_A + S} * X_A$	(1)
Strain B Density	$\frac{dX_B}{dt} = -D * X_B + \frac{S}{K_B + S} * \mu_{b\_B} * fHPr(AI1) * X_B$	(2)
Substrate Concentration	$\frac{dS}{dt} = D * (S_0 - S) - \frac{1}{Y_A}\frac{\mu_{b\_A} * S}{K_A + S} * X_A - \frac{1}{Y_B}\frac{\mu_{b\_B} * S}{K_B + S} * fHPr(AI1) * X_B$	(3)
AI-1 Concentration	$\frac{dAI1}{dt} = D * \left(AI1_{feed} - AI1\right)$	(4)
	$\frac{dAI1}{dt} = D * \left(AI1_{feed} - AI1\right) + AI1_{rate} * X_A$	(5)
	$\frac{dAI1}{dt} = -D * AI1 + fAI1(AI2) * X_A$	(6)
AI-2 Concentration	$\frac{dAI2}{dt} = -D * AI2 + AI2_{rate} * X_B$	(7)
**Where**		
Function for increased growth rate (caused by HPr) in Population B	$fHPr(AI1) = D + \frac{A - D}{\left(1 + \left(\frac{AI1}{C}\right)^B\right)^E}$ Where $A = 1$, $B = 1$, $C = 29$, $D = 2.1$, $E = 0.48$	
Function for rate of AI-1 production in Population A as a function of AI-2	$fAI1(AI2) = D + \frac{A - D}{\left(1 + \left(\frac{AI2}{C}\right)^B\right)^E}$ Where $A = 0$, $B = 1$, $C = 40$, $D = 2$, $E = 2$	

Simulation	Differential Equations
Co-culture Set 1 (Supplementary Figure 9b)	Equations (1), (2), (3), (4)
Co-culture Set 2 (Supplementary Figures 9c and 9e)	Equations (1), (2), (3), (5)
Co-culture Set 3 (Supplementary Figure 9d)	Equations (1), (2), (3), (6), (7)

Supplementary Table 5: Initial conditions and constants used in chemostat simulations.

Species	Description	Initial Condition
X_A	Population A concentration (OD600)	0.4
X_B	Population B concentration (OD600)	0.4
S	Substrate Concentration (g*L^{-1})	4
$AI1$	AI-1 Concentration (nM)	0
$AI2$	Supplemented AI-2 Concentration (μM)	0
t	Time (hr)	0
**Parameter**	**Description**	**Value**
D	Dilution rate (hr^{-1})	varied
S_0	Feed substrate concentration (g*L^{-1})	4
μ_{b_A}	Basal specific growth rate of Population A (hr^{-1}) (Used for simulation in Supplementary Figure 9b)	0.3
μ_{b_A}	Basal specific growth rate of Population A (hr^{-1}) (Used for simulations in Supplementary Figures 9c-9e)	0.27
μ_{b_B}	Basal specific growth rate of Population B (hr^{-1})	0.2
K_A & K_B	Substrate concentration for half max growth (g/L)	0.1
Y_A & Y_B	Yield (OD600 cells)/(g/L substrate)	0.45
$AI1_{rate}$	Rate of AI-1 production by Population A (nM*OD600^{-1}*hr^{-1})	1
$AI2_{rate}$	Rate of AI-2 production by Population B (μM*OD600^{-1}*hr^{-1})	10
$AI1_{feed}$	AI-1 in feed when controller is ON (nM)	10

Supplementary Table 6: Strains and Plasmids

Strains	Relevant Genotype	Source
E. coli		
W3110	K12 strain, wild type, λ-, F-, IN(*rrnD*-*rrnE*)1, *rph-1s*	Genetic Stock Center Yale University, New Haven, CT
TOP10	F- *mcrA* Δ(*mrr-hsd*RMS-*mcr*BC) Φ80*lac*ZΔM15 Δ*lac*X74 *rec*A1 *ara*D139 Δ(*araleu*)7697 *gal*U *gal*K *rps*L (StrR) *end*A1 *nup*G	Invitrogen
BL21	B strain, F-*ompT* [*dcm*][*lon*]*hsdS*(r_B-M_B-)*gal*	Novagen
BL21 luxS⁻	BL21 Δ*luxS*:: Kan	[2]
ZK126	W3110 Δ*lacU169 tna-2*	[3]
LW7	W3110 Δ*lacU169 tna-2* Δ*luxS*::Kan	[4]
PH01	W3110 Δ*lacU169 tna-2* Δ*ptsH*::Cm	[1]
PH02	W3110 Δ*lacU169 tna-2* Δ*luxS*::Kan Δ*ptsH*::Cm	[1]
PH03	W3110 Δ*lacU169 tna-2* Δ*ptsH*	This study
PH04	W3110 Δ*lacU169 tna-2* Δ*luxS* Δ*ptsH*	This study
CT104	W3110 *luxS*::Tcr, lsrFG-, W3110-derived *luxS*, *lsrFG* mutant strain	[5]
V. harveyi		
BB170	BB120 *luxN*::Tn5 (sensor 1- ,sensor 2+), Km^r	[6]

Plasmids	Description	Source
pET200	Cloning vector, containing *T7* promoter, Km^r	Invitrogen
pFZY1	*galK'-lacZYA* transcriptional fusion vector, Ap^r	[7]
pCT6	pFZY1 derivative, containing *lsrR* and *lsrR* promoter region fused with T7RPol, Ap^r	[8]
pSkunk-HPr	containing *ptsH* under tac promoter, streptomycin/spectomycin resistance	[1]
pLSR	pTS40 derivative, containing SpeI and PvuI restriction digestion sites, Cm^r	[9]
pT5G	eGFP under constitutive T5 promoter, Km^r	[10]
pTT01	pBR322, *soxR* gene and the overlapping divergent *soxR* and *soxS* promoters, phiLOV downstream of *soxS* promoter, Ap^r	[10]
pSox-LasI	pTT01 derivate, containing *lasI* under *soxS* promoter, Ap^r	This study
pAHL-Reporter_Red-Green (pAHL-sfGFP)	pET21a derivative, containing *sfGFP* under *lasI* promoter and *lasR* and *dsRedExpress2* under constitutive promoter, Ap^r	[11]
pLasI	pET200 derivate, containing *lasI* under *T7* promoter, Km^r	This study
pTac-HPr	pLSR derivate, containing *ptsH* under *tac* promoter, Cm^r	This study
pAHL-HPr	pAHL-Reporter_Red-Green derivative, containing *ptsH* under *lasI* promoter, Ap^r	This study
pAL105	*lasR*$^+$ *lasI::luxCDABE*; Tet^r p15A origin	[12]

Supplementary Table 7: Primers

Primer	Sequence
LasI-F	CACCATGATCGTACAAATTGGTCGGCGC
LasI-R	TCATGAAACCGCCAGTCGCTG
TacProm-PvuI-F	TGCATCGATCGTACGACTCACTATAGGGCGAATTCTG
Sox-R-GB	AATATCGATGATAAGCTGTCAAACATG
Sox-F-GB	AAGCTTAAATCTGCCTCTTTTCAG
pSoxLasI-F-GB	ACTGAAAAGAGGCAGATTTAAGCTTATGATCGTACAAATTGGTC
pSoxLasI-R-GB	CATGTTTGACAGCTTATCATCGATATTATGAAACCGCCAGT
HPr-SpeI-R	AGTCTAGACTAGTTTACTCGAGTTCCGCCATCAGTTTAACCAG
HPr-SpeI-F	TGCATACTAGTATGTTCCAGCAAGAAGTTACCG
HPr-SacI-R	TTCTTAGAGCTCTTACTCGAGTTCCGCCATC

Supplementary Notes

Supplementary Note 1

We estimated the growth rate of Population B and the AI-1 levels during the extended co-culture experiment (data points in **Figure 7d**) using a combination of the experimental data and the model. To estimate the growth of Population B, we first estimated the cell density of Population A over time. Assuming a constant specific growth rate for Population A (μ_{b_A}) and an initial inoculation density of X_{A_i}, we calculated the Population A density at 0, 3, 6 and 9 hours. X_{A_i} was determined using the average of the initial total OD (0.053) and the average of the initial "Fraction Population B" experimental data. We then calculated the Population B density at 0, 3, 6 and 9 hours using the calculated Population A density and the experimental "Fraction Population B" data. We plotted the natural log of the Population B density over time and fitted a 2nd order polynomial trend line through the data for each AI-2 concentration. We used the slope of the trend lines to estimate the growth rate of Population B over 9 hours in increments of 0.1 hours for each AI-2 concentration. For each three hour time segment (0-3 hr, 3-6 hr, and 6-9 hr), we calculated the average growth rate by averaging the values of each 0.1 hour increment. We normalized these values to μ_{b_B} in order to obtain relative growth rate. We used the model to determine corresponding average AI-1 levels. To do this, we modeled Population A using the adjusted model (see **Supplementary Note 2**) to determine the AI-1 level at 0.1 hour increments over the 9 hour experiment. We determined average AI-1 for each three hour segment and each AI-2 concentration. The average relative growth rate vs average AI-1 levels are plotted as data points in **Figure 7d**.

Supplementary Note 2

To model the extended co-culture experiments, including resuspension of the cells in fresh media containing AI-2, we used an adjusted model where additional assumptions were incorporated based on the experimental results. The experiment consisted of an initial co-culture inoculated into media with AI-2. At 3 and 6 hours, the culture was resuspended in fresh media. We note that to simulate each resuspension, the cell densities at the end of each three hour simulation were input back into the model along with the parameters for the fresh media (8 g/L glucose, supplemented AI-2 level, and 0 nM AI-1) and the simulation was restarted. For all simulations of the extended co-culture experiments, the total initial OD used at t = 0 was 0.053.

The first assumption used in the adjusted model was to account for the increased level of AI-1 produced by Population A with each resuspension. This increase likely occurred because the AI-1 synthase, LasI, did not immediately degrade each time the cells were resuspended. In the adjusted model, the rate of AI-1 produced (*fAI1*) was assumed to be the value of the *fAI1* function at the end of the prior three hours plus the value of the *fAI1* function calculated from the time and initial AI-2 level during the current simulation. The second change in the model was an adjustment to the AI-1 regulated growth rate (*fHPr*) in Population B. The *fHPr* logistic function had the form $f(x) = D + (A-D)/(1+(x/C)^B)^E$ as in the original model. In the adjusted model the constants used were A = 1, B = 1, C =3, and E = 0.48. We assumed the value of D to be a function of time. In the adjusted model D(t) = -0.27t + 3 where t ranges from 0 to 9 hours. This results in reduced AI-1 mediated increase in growth rate and decreased growth rate at later time periods. We also assumed that the value of the *fHPr* function did not immediately revert back to basal level when resuspended in media without AI-1 (presumably the protein HPr would still be present). To do this, the function *fHPr* for relative growth rate, used the AI-1 level from the end of the prior three hours ($AI1_{prior}$) until the current AI-1 level surpassed $AI1_{prior}$ at which point the current AI-1 level was used in the *fHPr* function.

Supplementary Note 3

The mathematical model can be used to investigate *in silico* how the strategies developed in this manuscript could be used to design co-culture systems with varied or controlled behaviors in continuous cultures. For instance, we designed three theoretical co-cultures of increasing complexity and simulated their behaviors in chemostats with or without a controller (**Supplementary Figure 8a**). In the first case (**Supplementary Figure 8b**), the co-culture (set 1) consists of one population that is not directly controlled (Population A) and one population where growth rate is a function of the AI-1 signal (Population B). An on-off (bang-bang) control scheme is used to control the culture composition at a programmed set point. The controller samples the culture composition hourly and compares the measured fraction of Population B to the set point. If the fraction of Population B is lower than the set point, an AI-1 feed pump is actuated, enabling a fixed concentration of AI-1 in the feed. The AI-1 increases the growth rate of Population B, driving the fraction of Population B up (closer to the set point). The AI-1 remains in the feed for the next 60 minutes (the sample frequency selected for these simulations), after which the controller samples the culture composition again. If the fraction is higher than the set point, AI-1 is no longer added to the feed. If it is still lower, AI-1 continues to be added in the feed. In the simulations depicted, eventually, the correct composition is reached and AI-1 is removed from the feed. At this point, AI-1 that has accumulated in the chemostat causes some overshoot of the set point while the dilution rate and removal of AI-1 (in the outlet) results in eventual decrease in Population B growth rate and return to the set point. This cycle continues and allows the culture composition to target the set point. Importantly, the minimum (without AI-1) and maximum (with AI-1) specific growth rates of Population B must span the specific growth rate of Population A for the system to work. In this way, the Population B growth rate can be increased or decreased as necessary so that neither population outgrows the other over time. We note that changing the dilution rate (depicted in **Supplementary Figure 8b**) results in a change in growth rates of both populations (as is typical of a chemostat) but the controller maintains the system at the set point. Also in this figure, we show that the controller allows for the set point to be changed or for the system to recover from perturbations.

We simulated a second case (co-culture set 2) without the controller. In this case (**Supplementary Figure 8c**), Population A produces AI-1 at a constant rate (per cell) and Population B growth rate is a function of AI-1. There is no AI-1 in the feed. Interestingly, a change in dilution rate causes an initial increase or decrease in AI-1 levels (due to increased or decreased removal of AI-1 in the feed) resulting in a change in Population B growth rate and a gradual change in culture composition. As the AI-1 producing population (Population A) decreases or increases, the AI-1 level approaches the steady state level, which is the same regardless of dilution rate. This steady state AI-1 level is the concentration of AI-1 at which the growth rates of both populations are identical. Thus, setting the dilution rate sets the culture composition and there is no set point for the fraction of Population B. The case in **Supplementary Figure 8d** shows similar behavior (co-culture set 3). Set 3 populations are the same as set 2, except that Population B produces AI-2 at a constant rate and Population A produces AI-1 at a rate that is a function of AI-2 level. Changing the dilution rate in this case also changes the steady state culture composition. This case results in higher levels of Population B for a set dilution rate than the case shown in **Supplementary Figure 8c** using co-culture set 2. In both of these examples, the culture composition is regulated autonomously by the cells and can only be changed by changing the dilution rate. In a sense, this condition is hands off and could be anticipated or otherwise designed in advance using the model. A user specified fraction is not achieved via this simple control process.

By extensions, in **Supplementary Figure 8e**, co-culture set 2 is now simulated in a chemostat with the on-off controller, adding an additional layer of control. Here, when the controller is off, the system reaches a specific composition at steady state (based on the dilution rate). Turning the controller on allows the system to target a higher set point, independent of the dilution rate. We note, however, a lower set point than what would naturally be achieved by the co-culture cannot be targeted since Population A is

producing a background level of AI-1. That is, the system is designed to operate within certain bounds and can only be fine-tuned within those bounds.

Several assumptions are made in these analyses that simplify the actual system. First, there are no delays in responses to signals. Removal of a signal results in an immediate decrease in the cell response to that signal (e.g. growth rate immediately decreases upon removal of AI-1). This type of response is a consequence of the simple Monod growth kinetics. Experimentally, incorporation of degradation tags on expressed proteins could result in quicker responses to changes in signal levels. Moreover, cell growth dynamics could be introduced as process lags. We have also assumed that the populations produce QS signals at constant rates independent of cell growth rate. However, the rate of AI-2 production in a chemostat actually changes as a function of the dilution rate[13]. Nevertheless, we suggest that the model offers insight into how co-cultures could be designed to operate in a continuous mode and how changes to the design may affect the outcome.

In sum, these scenarios provide a basis for how a cell-based autonomous controller system could be integrated into a reactor scheme that is operated based on user input. In all cases, one needs to provide a dilution rate. In some scenarios, this will define the composition irrespective of the genetic regulatory structure developed in this manuscript. In other words, although the genetic regulatory structure allows both populations to be maintained in continuous culture (something that would not normally happen), the engineered co-culture does not target a user-defined composition. In other scenarios, our autonomous controller system can be integrated with chemostats that do enable a user defined set point, including when simple on-off controllers provide an additional external input (e.g., AI-1) feed. We note, however, that it is unlikely that a composition sampling system and autoinducer feed would ever be implemented in a continuous system owing to the associated cost. That said, the cascaded control scheme in **Supplementary Figure 8e** suggests that the cell-based autonomous controller can be designed to operate within certain parameters and then adjusted within those parameters by addition of exogenous signals or inducers.

Simulink Version 8.7 (R2016a) was used for the chemostat simulations. The systems of ordinary differential equations (**Supplementary Tables 4 and 5**) were solved using the ode45 solver. To model the controller in Simulink, a chart block was used. The chart block was designed to take an input (the culture composition from the model) and report a "0" if the input was greater than the set point and a "1" if the input was less than the set point. A "1" value directed the model to add AI-1 to the chemostat feed. A "0" value directed the model to stop adding AI-1 to the feed. The sample time for the block was set to 1 hour so that the controller would only perform this check every 60 minutes.

Supplementary References

1. Ha, J.H. et al. Evidence of link between quorum sensing and sugar metabolism in Escherichia coli revealed via cocrystal structures of LsrK and HPr. *Sci Adv* **4**, eaar7063 (2018).
2. Fernandes, R. & Bentley, W.E. AI-2 biosynthesis module in a magnetic nanofactory alters bacterial response via localized synthesis and delivery. *Biotechnol Bioeng* **102**, 390-399 (2009).
3. Connell, N., Han, Z., Moreno, F. & Kolter, R. An E. coli promoter induced by the cessation of growth. *Mol Microbiol* **1**, 195-201 (1987).
4. Wang, L., Hashimoto, Y., Tsao, C.Y., Valdes, J.J. & Bentley, W.E. Cyclic AMP (cAMP) and cAMP receptor protein influence both synthesis and uptake of extracellular autoinducer 2 in Escherichia coli. *J Bacteriol* **187**, 2066-2076 (2005).
5. Servinsky, M.D. et al. Directed assembly of a bacterial quorum. *ISME J* **10**, 158-169 (2016).
6. Bassler, B.L., Greenberg, E.P. & Stevens, A.M. Cross-species induction of luminescence in the quorum-sensing bacterium Vibrio harveyi. *J Bacteriol* **179**, 4043-4045 (1997).
7. Koop, A.H., Hartley, M.E. & Bourgeois, S. A low-copy-number vector utilizing beta-galactosidase for the analysis of gene control elements. *Gene* **52**, 245-256 (1987).
8. Tsao, C.Y., Hooshangi, S., Wu, H.C., Valdes, J.J. & Bentley, W.E. Autonomous induction of recombinant proteins by minimally rewiring native quorum sensing regulon of *E. coli*. *Metab Eng* **12**, 291-297 (2010).
9. Hauk, P. et al. Insightful directed evolution of Escherichia coli quorum sensing promoter region of the lsrACDBFG operon: a tool for synthetic biology systems and protein expression. *Nucleic Acids Res* **44**, 10515-10525 (2016).
10. Tschirhart, T. et al. Electronic control of gene expression and cell behaviour in Escherichia coli through redox signalling. *Nat Commun* **8**, 14030 (2017).
11. Rhoads, M.K. et al. Modification and Assembly of a Versatile Lactonase for Bacterial Quorum Quenching. *Molecules* **23**, 341 (2018).
12. Lindsay, A. & Ahmer, B.M. Effect of sdiA on biosensors of N-acylhomoserine lactones. *J Bacteriol* **187**, 5054-5058 (2005).
13. DeLisa, M.P., Valdes, J.J. & Bentley, W.E. Mapping stress-induced changes in autoinducer AI-2 production in chemostat-cultivated Escherichia coli K-12. *J Bacteriol* **183**, 2918-2928 (2001).
