# Supplementary material for: Bacterial co-culture with cell signaling translator and growth controller modules for autonomously regulated culture composition
Source: Nat Commun. 2019 Sep 11;10:4129. doi: 10.1038/s41467-019-12027-6 (PMC6739400; doi:10.1038/s41467-019-12027-6)
Supplement: Supplementary file 3 — Reporting Summary [file 41467_2019_12027_MOESM3_ESM.pdf]

nature research

Corresponding author(s): William E. Bentley

Last updated by author(s): Jul 9, 2019

Reporting Summary

Nature Research wishes to improve the reproducibility of the work that we publish. This form provides structure for consistency and transparency in reporting. For further information on Nature Research policies, see Authors & Referees and the Editorial Policy Checklist.

Statistics

For all statistical analyses, confirm that the following items are present in the figure legend, table legend, main text, or Methods section.

n/a	Confirmed	
☐	☒	The exact sample size (n) for each experimental group/condition, given as a discrete number and unit of measurement
☐	☒	A statement on whether measurements were taken from distinct samples or whether the same sample was measured repeatedly
☒	☐	The statistical test(s) used AND whether they are one- or two-sided<br>*Only common tests should be described solely by name; describe more complex techniques in the Methods section.*
☒	☐	A description of all covariates tested
☐	☒	A description of any assumptions or corrections, such as tests of normality and adjustment for multiple comparisons
☐	☒	A full description of the statistical parameters including central tendency (e.g. means) or other basic estimates (e.g. regression coefficient) AND variation (e.g. standard deviation) or associated estimates of uncertainty (e.g. confidence intervals)
☒	☐	For null hypothesis testing, the test statistic (e.g. F, t, r) with confidence intervals, effect sizes, degrees of freedom and P value noted<br>*Give P values as exact values whenever suitable.*
☒	☐	For Bayesian analysis, information on the choice of priors and Markov chain Monte Carlo settings
☒	☐	For hierarchical and complex designs, identification of the appropriate level for tests and full reporting of outcomes
☒	☐	Estimates of effect sizes (e.g. Cohen's d, Pearson's r), indicating how they were calculated

Our web collection on statistics for biologists contains articles on many of the points above.

Software and code

Policy information about availability of computer code

Data collection	Olympus cellSens Standard 1.16 software was used for collecting microscopy images.
Data analysis	Fiji ImageJ 1.51 was used to count cells in microscopy images. The "batch process" option was used, where a custom macro can be used to process many images identically. For each image, the custom macro directed the software to set a specified threshold and count the cells using the "analyze particles" option. For bright field images (as opposed to fluorescent images), the "subtract rolling background" feature was also used prior to setting a threshold.<br>The MATLAB (Version R2016a) ode45 solver was used to solve the system of ordinary differential equations comprising the co-culture model. Results from the model were exported to Microsoft Excel in order to generate graphs. Simulink Version 8.7 (R2016a) was used to solve the system of ordinary differential equations for the chemostat models. The data from the chemostat simulations were exported to Matlab and the graphs were generated in Matlab.

For manuscripts utilizing custom algorithms or software that are central to the research but not yet described in published literature, software must be made available to editors/reviewers. We strongly encourage code deposition in a community repository (e.g. GitHub). See the Nature Research guidelines for submitting code & software for further information.

Data

Policy information about availability of data

All manuscripts must include a data availability statement. This statement should provide the following information, where applicable:

- Accession codes, unique identifiers, or web links for publicly available datasets
- A list of figures that have associated raw data
- A description of any restrictions on data availability

The data that support the findings of this study are available from the corresponding author upon reasonable request.

Field-specific reporting

Please select the one below that is the best fit for your research. If you are not sure, read the appropriate sections before making your selection.

- [x] Life sciences
- [ ] Behavioural & social sciences
- [ ] Ecological, evolutionary & environmental sciences

For a reference copy of the document with all sections, see nature.com/documents/nr-reporting-summary-flat.pdf

Life sciences study design

All studies must disclose on these points even when the disclosure is negative.

Sample size	For biological experiments which demonstrate effect of autoinducers on synthesis of autoinducer, growth rate, and on composition of co-cultures, at least 3 sets of experiments were run prior to the specific experiments depicted in our figures. That is, we ran campaigns of experiments and have depicted representative results from one complete set. Each set has its own internal controls. Our figure legends are clear relative to sample size and statistical significance. Generally, we expected large differences between experimental conditions and believe this way of representing these data is appropriate.
Data exclusions	No data were excluded from the analyses.
Replication	The figures in the main text and the supplemental section demonstrate the reproducibility of the system and the data. For instance, each cell line was tested in multiple experimental set ups. For instance, "controller" cells were tested both by addition of AI-1 and by addition of conditioned media containing AI-1. In all cases, experimental results supported the reproducibility of the system and the data.
Randomization	Randomization was not relevant to this study since the same overnight culture was used for all conditions in an experiment.
Blinding	Blinding was not relevant to this study. Use of software for counting cells in images avoided introduction of user bias.

Reporting for specific materials, systems and methods

We require information from authors about some types of materials, experimental systems and methods used in many studies. Here, indicate whether each material, system or method listed is relevant to your study. If you are not sure if a list item applies to your research, read the appropriate section before selecting a response.

Materials & experimental systems

n/a	Involved in the study	
☒	☐	Antibodies
☒	☐	Eukaryotic cell lines
☒	☐	Palaeontology
☐	☒	Animals and other organisms
☒	☐	Human research participants
☒	☐	Clinical data

Methods

n/a	Involved in the study	
☒	☐	ChIP-seq
☒	☐	Flow cytometry
☒	☐	MRI-based neuroimaging

Animals and other organisms

Policy information about studies involving animals; ARRIVE guidelines recommended for reporting animal research

Laboratory animals	The study did not involve animals. E. coli W3110 and W3110 derivate strains were used for experiments. E. coli strain TOP10 was used for cloning. All strains are listed and described in detail in the supplemental section.
Wild animals	The study did not involve wild animals.
Field-collected samples	The study did not involve samples collected from the field.
Ethics oversight	No ethical approval or guidance in the design of this study by an organization was required. Only commonly used E. coli strains were used in this study. All experiments were carried out in accordance with University of Maryland regulations.

Note that full information on the approval of the study protocol must also be provided in the manuscript.
