# Supplementary material for: Bacterial co-culture with cell signaling translator and growth controller modules for autonomously regulated culture composition
Source: Nat Commun. 2019 Sep 11;10:4129. doi: 10.1038/s41467-019-12027-6 (PMC6739400; doi:10.1038/s41467-019-12027-6)
Supplement: Supplementary file 4 — Description of Additional Supplementary Files [file 41467_2019_12027_MOESM4_ESM.pdf]

Description of Additional Supplementary Files

Title: Supplementary Software 1
Description: containing MATLAB code for batch model

Title: Supplementary Software 2
Description: containing MATLAB code for extended batch model
